# Sexual behaviour, STDs and risks for prostate cancer

**DOI:** 10.1054/bjoc.1999.0986

**Published:** 2000-01-18

**Authors:** R B Hayes, L M Pottern, H Strickler, C Rabkin, V Pope, G M Swanson, R S Greenberg, J B Schoenberg, J Liff, A G Schwartz, R N Hoover, J F Fraumeni

**Affiliations:** Division of Cancer Epidemiology and Genetics, National Cancer Institute, EPN 418, Bethesda, MD, 20892, USA; National Institutes of Health, Bethesda, USA; Centers for Disease Control and Prevention, Atlanta, GA, MD, USA; Michigan Cancer Foundation, Detroit, MI, USA; Department of Epidemiology, Rollins School of Public Health, Emory University, Atlanta, GA, USA; New Jersey State Department of Health, Trenton, NJ, USA

**Keywords:** epidemiology, prostatic neoplasms, racial aspects, sexual behaviour

## Abstract

A population-based case-control study was carried out among 981 men (479 black, 502 white) with pathologically confirmed prostate cancer and 1315 controls (594 black, 721 white). In-person interviews elicited information on sexual behaviour and other potential risk factors for prostate cancer. Blood was drawn for serologic studies in a subset of the cases (*n* = 276) and controls (*n* = 295). Prostate cancer risk was increased among men who reported a history of gonorrhoea or syphilis (odds ratio (OR) = 1.6; 95% confidence internal (CI) 1.2–2.1) or showed serological evidence of syphilis (MHA-TP) (OR = 1.8; 95% CI 1.0–3.5). Patterns of risk for gonorrhoea and syphilis were similar for blacks (OR = 1.7; 95% CI 1.2–2.2) and whites (OR = 1.6; 95% CI 0.8–3.2). Risks increased with increasing occurrences of gonorrhoea, rising to OR = 3.3 (95% CI 1.4–7.8) among subjects with three or more events (*P*_trend_= 0.0005). Frequent sexual encounters with prostitutes and failure to use condoms were also associated with increased risk. Syphilis, gonorrhoea, sex with prostitutes and unprotected sexual intercourse may be indicators of contact with a sexually transmissible factor that increases the risk of prostate cancer. © 2000 Cancer Research Campaign


*British Journal of Cancer* (2000) **82**(3), 718–725
© 2000 Cancer Research Campaign
DOI: 10.1054/ bjoc.1999.0986, available online at http://www.idealibrary.com on IDEAL®

### Sexual behaviour, STDs and risks for prostate cancer

**RB Hayes[1], LM Pottern[2], H Strickler[1], C Rabkin[1], V Pope[3], GM Swanson[4,*], RS Greenberg[5,†], JB Schoenberg[6], J Liff[5], AG Schwartz[4,‡], RN Hoover[1] and JF Fraumeni Jr[1]**

[1]Division of Cancer Epidemiology and Genetics, National Cancer Institute, EPN 418, Bethesda, MD 20892, USA; [2]Office of the Director, National Institutes of Health, Bethesda, MD, USA; [3]Centers for Disease Control and Prevention, Atlanta, GA, USA; [4]Michigan Cancer Foundation, Detroit, MI, USA; [5]Department of Epidemiology, Rollins School of Public Health, Emory University, Atlanta, GA, USA; [6]Formerly with the Special Epidemiology Program, New Jersey State Department of Health, Trenton, NJ, USA

**Summary** A population-based case-control study was carried out among 981 men (479 black, 502 white) with pathologically confirmed prostate cancer and 1315 controls (594 black, 721 white). In-person interviews elicited information on sexual behaviour and other potential risk factors for prostate cancer. Blood was drawn for serologic studies in a subset of the cases ($n$ = 276) and controls ($n$ = 295). Prostate cancer risk was increased among men who reported a history of gonorrhoea or syphilis (odds ratio (OR) = 1.6; 95% confidence internal (CI) 1.2–2.1) or showed serological evidence of syphilis (MHA-TP) (OR = 1.8; 95% CI 1.0–3.5). Patterns of risk for gonorrhoea and syphilis were similar for blacks (OR = 1.7; 95% CI 1.2–2.2) and whites (OR = 1.6; 95% CI 0.8–3.2). Risks increased with increasing occurrences of gonorrhoea, rising to OR = 3.3 (95% CI 1.4–7.8) among subjects with three or more events ($P_{trend}$ = 0.0005). Frequent sexual encounters with prostitutes and failure to use condoms were also associated with increased risk. Syphilis, gonorrhoea, sex with prostitutes and unprotected sexual intercourse may be indicators of contact with a sexually transmissible factor that increases the risk of prostate cancer. © 2000 Cancer Research Campaign

**Keywords:** epidemiology; prostatic neoplasms; racial aspects; sexual behaviour

In the US, black men are diagnosed with prostate cancer about 70% more often than white men (blacks, 234.4 cases per 100 000; whites, 135.3 per 100 000); prostate cancer mortality is about 130% greater among black than white men (blacks, 55.5 deaths per 100 000; whites, 23.8 per 100 000) (National Cancer Institute, 1997). The causes of prostate cancer are only partially understood and the reasons for the racial differentials in risk are unknown.

Beginning in the 1970s, a series of epidemiological investigations found that aspects of sexual behaviour are associated with risk for prostate cancer, suggesting the role of sex hormones or sexually transmitted diseases (STDs) in the aetiology of prostate cancer (Ross and Schottenfeld, 1996). The studies, however, were generally small or had other methodological limitations, so that the significance of the findings and their interpretation were uncertain (Nomura and Kolonel, 1991; Ross and Schottenfeld, 1996; Pienta and Esper, 1993).

Although several STDs occur more frequently among US blacks than whites (Division of STD Prevention, 1997), only limited attention has been given to the differential impact of STDs or other aspects of sexual behaviour on prostate cancer risk in these populations (Jackson et al, 1980; Ross et al, 1987). We carried out a large population-based case–control study of prostate cancer among black and white men in three areas of the US. Associations with sexual activity and possible exposure to sexually transmissible agents were evaluated through in-person interviews. Serological investigations for selected STDs were carried out in a subset of the study group.

*Received 8 December 1998*
*Revised 13 April 1999*
*Accepted 28 April 1999*

*Correspondence to:* RB Hayes

### METHODS

### Study design

This case–control study of prostate cancer among US blacks and whites was conducted as one component of a multi-site study that also included cancers of the oesophagus and pancreas, and multiple myeloma. The investigation received Institutional Review Board approval. Study subjects resided in geographic areas covered by the population-based cancer registry of the Georgia Center for Cancer Statistics (Fulton and DeKalb counties), the Metropolitan Detroit Cancer Surveillance System (Wayne, Oakland and Macomb counties), and the New Jersey State Cancer Registry (ten counties).

### Questionnaire-based study

Cases for this study were men aged 40–79, identified from pathology and outpatient records at hospitals covered by these registries, and newly diagnosed with pathologically confirmed prostate cancer between 1 August, 1986 and 30 April, 1989. Identified cases were included for study based upon an age- and race-stratified sampling scheme to ensure representation of both blacks and whites in a broad age range. Study cases were classified by tumour stage (localized or advanced [including regional/ distant]) and grade (well, moderate, or undifferentiated). Population controls were selected in the three geographic areas

*Present address: College of Human Medicine, Michigan State University, East Lansing, MI, USA

†Present address: Medical University of South Carolina, Charleston, SC, USA

‡Present address: MCP-Hahnemann School of Medicine, Allegheny University of the Health Sciences, Pittsburgh, PA, USA

**Table 1** Study subjects

| | Controls | | | Cases | | |
|---|---|---|---|---|---|---|
| | **Black** | **White** | **Total** | **Black** | **White** | **Total** |
| Interviewed subjects | 594 | 721 | 1315 | 479 | 502 | 981 |
| Eligible for serologic studies | 213 | 254 | 467 | 234 | 251 | 485 |
| Blood drawn | 137 | 158 | 295 | 127 | 149 | 276 |

proportional to the expected age, sex and race distribution of the combined cases for the four cancer sites. Controls younger than 65 years of age were selected by the Waksberg method of random digit dialling (RDD) (Waksberg, 1978); older controls were selected by random sampling from the computerized records of the Health Care Financing Administration.

In total, 1292 cases and 1767 controls were identified for study. In-person interviews were conducted for the cases and controls, usually in their homes. Prostate cancer cases and male controls were questioned about a number of factors, including demographics, height and weight, occupational history, family history of cancer, history of sexually transmitted disease, sexual behaviour, dietary intake, and tobacco and alcohol use. A socio-economic status score (modified from Green, 1970) was derived from review of the reported usual occupation. Interviews were obtained for 988 cases (76%; black = 78%, white = 75%) and 1336 controls (76%; black = 77%, white = 74%). After accounting for non-response in the initial phase of screening for eligibility among RDD contacts, the response rate in controls was 70%. Six cases and six controls were dropped from the analysis due to incomplete interviews. Sixteen subjects (one case, 15 controls) were excluded due to a prior history of prostate cancer. The final study group for analysis of interview data consisted of 981 cases (479 black, 502 white) and 1315 controls (594 black, 721 white) (Table 1). Distributions of selected variables in this study group are shown in Table 2.

### Laboratory-based study

Cases of prostate cancer were eligible for blood collection, which occurred about 12 weeks after diagnosis, if they had not had chemotherapy, radiation therapy, treatment with hormone-related drugs, or orchiectomy prior to blood collection. A series of controls was also selected for blood collection based on the age and race distribution of the eligible cases. Venous blood (50 ml) was drawn and aliquoted to serum, plasma and red blood cell/buffy coat fractions. Blood products were stored in 1 ml vials at –70°C. Among interviewed subjects, blood samples were successfully drawn for serological assays (syphilis and human papillomavirus (HPV)) from 276 (57%) of the eligible cases and 295 (63%) of the eligible controls (Table 1).

Specimens were tested in the microhaemagglutination assay for antibodies to *Treponema pallidum* (MHA-TP) (Kennedy, 1990). Serum antibodies to human papillomavirus (HPV-16) were assayed by a validated enzyme-linked immunosorbent assay (ELISA), with positive reactivity defined by previously determined optical density cut-off values (Strickler et al, 1997, 1999). Approximately 85% of

**Table 2** Selected characteristics of interviewed prostate cancer cases and controls.

| | US blacks | | US whites | |
|---|---|---|---|---|
| **Characteristic** | **Cases** | **Controls** | **Cases** | **Controls** |
| Age | | | | |
| 40–59 | 124 | 228 | 164 | 322 |
| 60–69 | 185 | 183 | 157 | 220 |
| 70 + | 170 | 183 | 181 | 179 |
| Study area | | | | |
| Atlanta | 88 | 124 | 103 | 158 |
| Detroit | 180 | 246 | 223 | 268 |
| New Jersey | 211 | 224 | 176 | 295 |
| Education | | | | |
| 0–8th grade | 199 | 215 | 55 | 89 |
| 9th–11th grade | 118 | 133 | 87 | 97 |
| 12th + | 161 | 246 | 359 | 529 |
| Alcohol, drinks per week | | | | |
| Never | 94 | 133 | 90 | 150 |
| ≤7 | 96 | 126 | 136 | 213 |
| 8–21 | 113 | 168 | 140 | 197 |
| 22–56 | 119 | 118 | 92 | 124 |
| ≥57 | 54 | 48 | 42 | 37 |
| Cigarette use | | | | |
| Never used tobacco | 88 | 116 | 86 | 149 |
| Current cigarette use | 161 | 221 | 116 | 177 |
| Former cigarette use | 189 | 199 | 243 | 319 |
| Cancer stage | | | | |
| Localized | 268 | | 312 | |
| Regional | 49 | | 59 | |
| Distant | 125 | | 73 | |
| Unknown | 26 | | 42 | |
| Total subjects | 479 | 594 | 502 | 721 |

© 2000 Cancer Research Campaign 

individuals who have been infected with *T. pallidum* will maintain circulating antibodies that are detectable by the MHA-TP for a lifetime (Haas et al, 1990). HPV capsid antibodies are also thought to exhibit long-term persistence (Carter et al, 1996).

### Statistical analysis

Odds ratios for prostate cancer were estimated by unconditional logistic regression (Breslow and Day, 1980). Adjustments for age, study site (Atlanta, Detroit, New Jersey), and, where appropriate, race were included in all logistic models. All categories were defined with common cut points for blacks and whites.

### RESULTS

### Interview study

Prostate cancer risk was increased among men who had a history of gonorrhoea or syphilis (OR = 1.6) (Table 3). Risks increased with increasing number of occurrences of gonorrhoea (*P* for trend = 0.0005), rising to odds ratio (OR) = 3.3 among men with three or more events. The patterns of risk were similar for blacks and whites; however, these conditions were reported more commonly among blacks (18.7% vs 2.5% in controls). Greater risk was associated with first episodes of gonorrhoea at 20–29 years of age, as compared to first episodes at younger or older ages. Risks were similarly elevated among men reporting gonorrhoea only or syphilis only, but the risks were highest among men reporting both conditions. Risks associated with these diseases were similar for all cases combined and for those with advanced (regional/distant) stage (data not shown).

The risks associated with a history of syphilis or gonorrhea were highest among men less than 60 years of age at prostate cancer diagnosis (blacks, 39 cases, OR = 2.2, 95% confidence interval CI 1.3–3.6; whites, nine cases, OR = 1.8, 95% CI 0.7–4.5). The risks were less pronounced among black men at age 60–69 years (46 cases, OR = 1.5, 95% CI 0.9–2.4) and at age 70+ years (41 cases, OR = 1.4, 95% CI 0.8–2.4), while no excess risk was found among white men at age 60–69 years (four cases, OR = 1.0, 95% CI 0.3–4.0) or at age 70+ years (three cases, OR = 0.9, 95% CI 0.2–4.4). Among blacks, the greatest risks were found in Atlanta (31 cases, OR = 7.3; 95% CI 3.2–16.5), with lower risks in New Jersey (42 cases, OR = 1.5; 95% CI 0.9–2.4) and Detroit (53 cases, OR = 1.1; 95% CI 0.7–1.7). Among whites, the corresponding ORs for the three study sites were 1.1 (four cases, 95% CI 0.3–4.2), 1.9 (five cases, 95% CI 0.5–6.8), and 1.2 (seven cases, 95% CI 0.4–3.5).

Although syphilis and gonorrhoea were reported more often among men of lower SES (14% of controls) than among men of medium (7.3% of controls) or high SES (4.7% of controls), risks of prostate cancer associated with these disorders were similar for men of low (95 cases, OR = 1.5, 95% CI 1.1–2.1), medium (40 cases, OR = 1.6, 95% CI 1.0–2.8), or high SES (seven cases, OR = 1.7, 95% CI 0.6–5.4). The association with history of syphilis or gonorrhoea was also not changed by statistical adjustment for education. We previously found associations of prostate cancer in this study with family history of prostate cancer (Hayes et al, 1995), heavy alcohol use (Hayes et al, 1997), and intake of fat from animal sources (Hayes et al, 1999), and weaker associations with cigarette use (Hayes et al, 1994) and vasectomy (Hayes et al, 1993); the association with history of syphilis or gonorrhoea was independent of these factors.

**Table 3** Risk of prostate cancer among US blacks and whites, by history of sexually transmitted disease

| | US blacks | | | | US whites | | | | Total | |
|---|---|---|---|---|---|---|---|---|---|---|
| Characteristic | Controls | Cases | OR[a] | 95% CI | Controls | Cases | OR[a] | 95% CI | OR[b] | 95% CI |
| History of gonorrhoea | | | | | | | | | | |
| Never | 485 | 362 | 1.0 | Referent | 693 | 486 | 1.0 | Referent | 1.0 | Referent |
| Ever | 103 | 115 | 1.6 | 1.2–2.2 | 18 | 15 | 1.4 | 0.7–2.9 | 1.5 | 1.1–2.0 |
| (%) ever | (17.5) | (24.1) | | | (2.5) | (3.0) | | | | |
| Frequency of gonorrhoea (referent: never) | | | | | | | | | | |
| 1 time | 68 | 68 | 1.4 | 1.0–2.1 | 12 | 12 | 1.6 | 0.7–3.7 | 1.4 | 1.0–1.9 |
| 2 | 23 | 26 | 1.7 | 0.9–3.1 | 4 | 2 | 1.0 | 0.2–5.5 | 1.5 | 0.9–2.6 |
| 3 + | 8 | 19 | 3.5 | 1.4–8.3 | | | | | 3.3 | 1.4–7.8 |
| *P* for trend | | | 0.0003 | | | | 0.45 | | 0.0005 | |
| Age first had gonorrhoea (referent: never) | | | | | | | | | | |
| <20 years | 45 | 38 | 1.3 | 0.8–2.2 | 2 | 5 | 5.1 | 1.0–26.8 | 1.4 | 0.9–2.2 |
| 20–29 | 42 | 67 | 2.2 | 1.4–3.4 | 7 | 6 | 1.3 | 0.4–4.2 | 2.0 | 1.4–3.0 |
| 30 + | 13 | 10 | 1.0 | 0.4–2.3 | 8 | 4 | 0.8 | 0.2–2.9 | 0.9 | 0.4–1.8 |
| History of syphilis | | | | | | | | | | |
| Never | 576 | 451 | 1.0 | Referent | 711 | 500 | 1.0 | Referent | 1.0 | Referent |
| Ever | 12 | 26 | 2.4 | 1.2–4.9 | 1 | 1 | 2.8 | 0.2–49.1 | 2.6 | 1.3–5.1 |
| (%) ever | (2.0) | (5.5) | | | (0.1) | (0.2) | | | | |
| History of syphilis or gonorrhoea | | | | | | | | | | |
| Never | 478 | 351 | 1.0 | Referent | 693 | 485 | 1.0 | Referent | 1.0 | Referent |
| Syphilis only | 7 | 11 | 1.8 | 0.7–4.7 | 0 | 1 | – | | 2.2 | 0.9–5.8 |
| Gonorrhoea only | 98 | 100 | 1.5 | 1.1–2.1 | 17 | 15 | 1.5 | 0.7–3.1 | 1.4 | 1.1–1.9 |
| Both | 5 | 15 | 3.9 | 1.4–11.0 | 1 | 0 | – | | 3.3 | 1.3–8.7 |
| Syphilis or gonorrhea | 110 | 126 | 1.7 | 1.2–2.2 | 18 | 16 | 1.6 | 0.8–3.2 | 1.6 | 1.2–2.1 |
| (%) either condition | (18.7) | (26.4) | | | (2.5) | (3.2) | | | | |

[a]Odds ratios, adjusted for age and study site. [b]Odds ratios, adjusted for age, study site, and race.

 *© 2000 Cancer Research Campaign*

**Table 4** Risk of prostate cancer among US blacks and whites, by selected characteristics

| Characteristic | US blacks | | | US whites | | | Total | |
|---|---|---|---|---|---|---|---|---|
| | Cases | OR[a] | 95% CI | Cases | OR[a] | 95% CI | OR[b] | 95% CI |
| Number of marriages | | | | | | | | |
| Never | 25 | 2.2 | 1.1–4.4 | 17 | 1.0 | 0.5–2.0 | 1.4 | 0.9–2.2 |
| 1 | 251 | 1.0 | Referent | 363 | 1.0 | Referent | 1.0 | Referent |
| 2 | 145 | 1.4 | 1.1–2.0 | 87 | 1.2 | 0.9–1.6 | 1.3 | 1.1–1.6 |
| 3+ | 35 | 0.9 | 0.5–1.4 | 23 | 1.8 | 1.0–3.4 | 1.0 | 0.7–1.5 |
| *P* for trend (excludes never married) | 0.42 | | | 0.05 | | 0.10 | | |
| Number of children | | | | | | | | |
| None | 86 | 1.0 | Referent | 67 | 1.0 | Referent | 1.0 | Referent |
| 1 | 67 | 1.1 | 0.7–1.7 | 56 | 0.9 | 0.6–1.5 | 1.0 | 0.7–1.4 |
| 2–3 | 148 | 0.8 | 0.6–1.2 | 251 | 0.9 | 0.6–1.3 | 0.9 | 0.7–1.1 |
| 4–5 | 96 | 0.9 | 0.6–1.4 | 93 | 0.9 | 0.6–1.3 | 0.9 | 0.7–1.2 |
| 6 + | 77 | 0.8 | 0.5–1.2 | 34 | 1.0 | 0.5–1.7 | 0.9 | 0.6–1.2 |
| *P* for trend | | 0.26 | | | 0.72 | | 0.32 | |
| Number of female sexual partners | | | | | | | | |
| <5 | 88 | 1.0 | Referent | 263 | 1.0 | Referent | 1.0 | Referent |
| 5–9 | 81 | 1.1 | 0.7–1.6 | 86 | 1.5 | 1.1–2.2 | 1.3 | 1.0–1.7 |
| 10–19 | 83 | 1.2 | 0.8–1.8 | 52 | 1.1 | 0.7–1.6 | 1.2 | 0.9–1.5 |
| 20–39 | 72 | 1.4 | 0.9–2.2 | 40 | 1.4 | 0.9–2.2 | 1.4 | 1.1–2.0 |
| 40 + | 83 | 1.2 | 0.8–1.8 | 28 | 1.2 | 0.7–2.0 | 1.2 | 0.9–1.6 |
| *P* for trend | | 0.27 | | | 0.29 | | 0.17 | |
| Sexual encounters with prostitutes | | | | | | | | |
| Never | 277 | 1.0 | Referent | 360 | 1.0 | Referent | 1.0 | Referent |
| <5 | 58 | 1.1 | 0.7–1.6 | 55 | 1.2 | 0.8–1.8 | 1.1 | 0.8–1.4 |
| 5–9 | 21 | 1.0 | 0.5–1.9 | 12 | 1.3 | 0.6–2.8 | 1.1 | 0.7–1.8 |
| 10–19 | 25 | 1.8 | 0.9–3.4 | 17 | 1.1 | 0.6–2.2 | 1.4 | 0.9–2.2 |
| 20–39 | 10 | 0.8 | 0.3–1.8 | 5 | 1.6 | 0.4–6.5 | 1.0 | 0.5–2.0 |
| 40 + | 17 | 1.6 | 0.7–3.5 | 15 | 4.2 | 1.5–11.9 | 2.3 | 1.3–4.2 |
| *P* for trend | | 0.27 | | | 0.004 | | 0.007 | |
| Years used a condom | | | | | | | | |
| Never | 322 | 1.0 | Referent | 352 | 1.0 | Referent | 1.0 | Referent |
| 1–10 | 43 | 1.0 | 0.7–1.7 | 30 | 1.1 | 0.7–1.9 | 1.1 | 0.8–1.6 |
| 11–20 | 10 | 0.4 | 0.2–0.9 | 31 | 1.8 | 1.0–3.1 | 1.0 | 0.6–1.6 |
| 21–30 | 16 | 0.9 | 0.4–1.8 | 24 | 0.7 | 0.4–1.2 | 0.8 | 0.5–1.2 |
| 30 + | 13 | 0.4 | 0.2–1.0 | 21 | 0.7 | 0.4–1.2 | 0.6 | 0.4–0.9 |
| *P* for trend | | 0.02 | | | 0.20 | | 0.009 | |
| Use of condoms with prostitutes | | | | | | | | |
| Never visited prostitutes | 277 | 1.0 | Referent | 360 | 1.0 | Referent | 1.0 | Referent |
| Usually used condoms | 47 | 1.0 | 0.6–1.5 | 36 | 1.1 | 0.7–1.7 | 1.0 | 0.7–1.4 |
| Usually didn't use condoms | 87 | 1.4 | 0.9–1.9 | 68 | 1.6 | 1.1–2.3 | 1.4 | 1.1–1.8 |
| *P* for trend | | 0.13 | | | 0.03 | | 0.01 | |

[a]Odds ratios, adjusted for age and study site. [b]Odds ratios, adjusted for age, study site and race.

Long-term use of condoms was associated with decreased risk of prostate cancer (Table 4). Risks were increased among men who had frequent encounters with prostitutes or who failed to use condoms with prostitutes. Number of sexual partners ($P_{trend}$ < 0.0001) and frequency of encounters with prostitutes ($P_{trend}$ < 0.0001) were strongly associated with a reported history of syphilis or gonorrhoea (data not shown). However, the number of sexual partners was unrelated to prostate cancer risk. Risks were elevated among never-married black men (OR = 2.2; 95% CI 1.1–4.4) and among white men who had three or more marriages (OR = 1.8; 95% CI 1.0–3.4). Number of children was unrelated to risk. When assessed together in a multivariate model, risks for prostate cancer associated with history of syphilis or gonorrhoea, sexual encounters with prostitutes, years of condom use and number of sexual partners remained substantially unchanged.

No clear patterns of risk were associated with age at first sexual intercourse. Although the frequency of intercourse was not consistently associated with risk, there was a suggestive relationship in some age groups, notably with frequency of intercourse in the fifth decade (Table 5). However, many subjects failed to report on frequency of intercourse.

### Serological study

Prostate cancer risk was increased among men with serologic evidence of infection with *T. pallidum* (OR = 1.8; 95% CI 1.0–3.5), particularly among blacks (Table 6). The evidence linking HPV-16 serology to prostate cancer was weaker (OR = 1.4; 95% CI 0.7–2.8), and derived mainly from excess risks among white men. Syphilis serology was associated with reported number of sexual partners ($P_{trend}$ = 0.009) and with frequency of encounters with prostitutes ($P_{trend}$ = 0.03). HPV serology, however, was not clearly related to these factors ($P_{trend}$ = 0.14 and $P_{trend}$ = 0.49, respectively). Serological evidence of syphilis (9.9% in black and

© 2000 Cancer Research Campaign

**Table 5** Risk of prostate cancer among US blacks and whites, by patterns of sexual intercourse

| Characteristic | US blacks | | | US whites | | | Total | |
|---|---|---|---|---|---|---|---|---|
| | Cases | OR[a] | 95% CI | Cases | OR[a] | 95% CI | OR[b] | 95% CI |
| Age of first sexual intercourse | | | | | | | | |
| <16 | 166 | 1.0 | Referent | 67 | 1.0 | Referent | 1.0 | Referent |
| 16–19 | 208 | 0.8 | 0.6–1.1 | 207 | 0.8 | 0.5–1.2 | 0.8 | 0.7–1.0 |
| 20–24 | 35 | 0.8 | 0.5–1.4 | 134 | 0.6 | 0.4–1.0 | 0.7 | 0.5–1.9 |
| 25–45 | 4 | 1.1 | 0.2–4.9 | 60 | 0.8 | 0.5–1.3 | 0.9 | 0.6–1.3 |
| *P* for trend | | 0.34 | | | 0.27 | | 0.18 | |
| Frequency of sexual intercourse in 20 s | | | | | | | | |
| <1/week | 48 | 1.0 | Referent | 84 | 1.0 | Referent | 1.0 | Referent |
| 1–2/week | 101 | 1.0 | 0.6–1.3 | 127 | 1.1 | 0.7–1.6 | 1.0 | 0.8–1.4 |
| >2–4/week | 132 | 1.5 | 1.0–2.4 | 146 | 1.2 | 0.8–1.8 | 1.3 | 1.0–1.8 |
| >4/week | 104 | 1.4 | 0.9–2.3 | 84 | 0.9 | 0.6–1.4 | 1.1 | 0.8–1.5 |
| unknown | 94 | 1.0 | 0.6–1.7 | 61 | 0.5 | 0.3–0.8 | 0.7 | 0.5–1.0 |
| *P* for trend | | 0.09 | | | 0.57 | | 0.52 | |
| Frequency of sexual intercourse in 30 s | | | | | | | | |
| <1/week | 25 | 1.0 | Referent | 31 | 1.0 | Referent | 1.0 | Referent |
| 1–2/week | 119 | 1.2 | 0.7–2.2 | 136 | 0.9 | 0.5–1.5 | 1.1 | 0.7–1.5 |
| >2–4/week | 151 | 1.7 | 1.0–3.0 | 186 | 1.2 | 0.7–2.1 | 1.4 | 1.0–2.1 |
| >4/week | 89 | 1.6 | 0.9–2.8 | 87 | 1.1 | 0.7–2.0 | 1.3 | 0.9–2.0 |
| unknown | 95 | 1.2 | 0.7–2.2 | 62 | 0.5 | 0.3–0.8 | 0.8 | 0.5–1.2 |
| *P* for trend | | 0.23 | | | 0.31 | | 0.12 | |
| Frequency of sexual intercourse in 40 s | | | | | | | | |
| <1/week | 35 | 1.0 | Referent | 53 | 1.0 | Referent | 1.0 | Referent |
| 1–2/week | 153 | 1.2 | 0.7–1.9 | 178 | 0.8 | 0.5–1.2 | 0.9 | 0.7–1.3 |
| >2–4/week | 145 | 1.9 | 1.1–3.1 | 159 | 1.1 | 0.7–1.6 | 1.4 | 1.0–1.9 |
| >4/week | 49 | 1.2 | 0.7–2.2 | 53 | 1.3 | 0.8–2.2 | 1.2 | 0.8–1.7 |
| unknown | 97 | 1.2 | 0.7–2.0 | 59 | 0.4 | 0.2–0.6 | 0.7 | 0.5–1.0 |
| *P* for trend | | 0.48 | | | 0.09 | | 0.10 | |
| Frequency of sexual intercourse in 50 s | | | | | | | | |
| <1/week | 78 | 1.0 | Referent | 96 | 1.0 | Referent | 1.0 | Referent |
| 1–2/week | 203 | 1.1 | 0.8–1.6 | 213 | 1.1 | 0.8–1.6 | 1.1 | 0.9–1.4 |
| >2/week | 93 | 1.3 | 0.9–2.0 | 106 | 1.6 | 1.1–2.4 | 1.5 | 1.1–2.0 |
| unknown | 105 | 1.0 | 0.7–1.5 | 87 | 0.5 | 0.4–0.8 | 0.8 | 0.6–1.0 |
| *P* for trend | | 0.20 | | | 0.01 | | 0.005 | |
| Frequency of sexual intercourse in 60 s (limited to men 60 + years of age) | | | | | | | | |
| none | 30 | 1.0 | Referent | 61 | 1.0 | Referent | 1.0 | Referent |
| <1/month | 70 | 1.0 | 0.5–1.9 | 47 | 0.4 | 0.2–0.7 | 0.6 | 0.4–0.9 |
| <1/week | 33 | 0.9 | 0.4–1.8 | 35 | 0.5 | 0.3–1.0 | 0.6 | 0.4–1.0 |
| 1–2/week | 116 | 1.0 | 0.6–1.8 | 122 | 0.7 | 0.4–1.1 | 0.8 | 0.5–1.1 |
| >2/week | 23 | 0.6 | 0.3–1.3 | 23 | 0.5 | 0.3–1.1 | 0.5 | 0.3–0.9 |
| unknown | 83 | 0.9 | 0.5–1.7 | 50 | 0.3 | 0.2–0.5 | 0.5 | 0.3–0.7 |
| *P* for trend | | 0.17 | | | 0.74 | | 0.26 | |

[a]Odds ratios, adjusted for age and study site. [b]Odds ratios, adjusted for age, study site and race. *P* for trend excludes unknowns.

3.2% in white controls, Table 6) was more common than self-reports (2% in black and 0.1% in white controls, Table 3). Among subjects who replied to the question on history of syphilis and for whom serology results were available (268 cases, 280 controls), three (23%) of 13 reports of syphilis were confirmed by MHA-TP serology, while 42 (7.9%) of 535 subjects who reported no history of syphilis were serology-positive.

### DISCUSSION

Our large population-based case–control study among US blacks and whites revealed an elevated risk of prostate cancer among men with a history of gonorrhoea or syphilis. Although STDs are not considered an established risk factor for prostate cancer (Nomura and Kolonel, 1991; Pienta and Esper, 1993; Ross and Schottenfeld, 1996), the results from several (Steele et al, 1971; Krain, 1974; Heshmat et al, 1975; Lees et al, 1985; Mishina et al, 1985; Mandel and Schuman, 1987; Ross et al, 1987; Honda et al, 1988), but not all previous studies (Wynder et al, 1971) are consistent with our findings. We also observed a greater prevalence of antibodies to *T. pallidum* among prostate cancer cases than controls, suggesting that recall or reporting bias can not entirely explain the relationship with STDs observed in retrospective interview studies.

The role of sexually transmitted factors in our study is suggested also by the excess risks of prostate cancer associated with frequent encounters with prostitutes and with failure to use condoms. Although some investigations have reported an

 © 2000 Cancer Research Campaign

**Table 6** Serological characteristics and risk of prostate cancer among US blacks and whites

| | US blacks | | | | US whites | | | | Total | |
|---|---|---|---|---|---|---|---|---|---|---|
| **Characteristic** | **Controls** | **Cases** | **OR[a]** | **95% CI** | **Controls** | **Cases** | **OR[a]** | **95% CI** | **OR[b]** | **95% CI** |
| Antibodies to syphillis | | | | | | | | | | |
| Non-reactive | 118 | 98 | 1.0 | Referent | 149 | 143 | 1.0 | Referent | 1.0 | Referent |
| Minimally reactive | 0 | 1 | – | | 1 | 0 | – | | 1.2 | 0.1–19.4 |
| Reactive | 13 | 26 | 2.2 | 1.0–4.6 | 5 | 3 | 0.8 | 0.2–3.5 | 1.8 | 1.0–3.5 |
| (%) reactive | (9.9) | (20.8) | | | (3.2) | (2.1) | | | | |
| Antibodies to HPV 16 | | | | | | | | | | |
| Negative | 120 | 116 | 1.0 | Referent | 144 | 132 | 1.0 | Referent | 1.0 | Referent |
| Indeterminate | 5 | 3 | 0.7 | 0.1–3.1 | 5 | 4 | 0.8 | 0.2–3.3 | 0.8 | 0.3–2.1 |
| Positive | 8 | 8 | 1.0 | 0.3–2.8 | 7 | 11 | 1.8 | 0.7–4.9 | 1.4 | 0.7–2.8 |
| (%) positive | (6.3) | (6.3) | | | (4.6) | (7.5) | | | | |

[a]Odds ratios, adjusted for age and study site. [b]Odds ratios, adjusted for age, study site, and race.

increased risk associated with number of sexual partners (Steele et al, 1971; Krain, 1974; Anderson et al, 1996), no relationship was seen in our study as well as others (Rotkin, 1977; Mandel and Schuman, 1987; Honda et al, 1988; La Vecchia et al, 1993). It is unclear why excess risks have been associated with a history of STDs but not consistently with number of sexual partners, raising the possibility that STD-associated prostate cancer is confined to a subset of the sexually active population, in which ecological factors as well as individual behaviours influence transmission of an infectious agent (Koopman and Longini, 1994; Brunham and Plummer, 1990).

Gonorrhoea and syphilis were reported substantially more often among blacks (18.7%) than whites (2.5%) in our study, consistent with reports from national surveillance data systems (Nakashima et al, 1996; Division of STD Prevention, 1997). However, the fraction of prostate cancer associated with syphilis or gonorrhoea (9.5% in blacks, 0.7% in whites) would account (assuming a causal relation) for a relatively small per cent of the black-white difference in cancer rates.

Since gonorrhoea and syphilis may be sentinels for a sexually transmitted infectious agent that contributes to the aetiology of prostate cancer, the risks we observed for STDs would only partially reflect the true risks associated with the putative agent. For example, only after sensitive and specific assays were developed for HPV did the relatively modest risks for cervical intra-epithelial neoplasia associated with sexual activity (two- to fourfold) translate to the substantial (50-fold) risks now established for cervical disease due to specific HPV subtypes (Schiffman et al, 1993). Although estimates of relative risk for prostate cancer were fairly consistent when measured by various definitions of sexually transmitted disease, population attributable risks remain uncertain as the prevalence estimates for STDs in our study varied substantially by disease type and by whether the method of ascertainment was by questionnaire or serology. Both questionnaire and serological measures are subject to measurement error (Romanowski et al, 1991; Anderson et al, 1994).

Although subgroup analyses in our study were limited by small numbers, the risks associated with history of STDs were greatest among younger men (< 60 years of age) and among black men in Atlanta. The age effect may indicate differential susceptibility or perhaps greater misclassification of these conditions among older men. More intriguing is the geographic variation in view of the high prevalence of gonorrhoea and syphilis in the southeastern States (Thomas et al, 1996; Kilmarx et al, 1997), as well as the elevated prostate cancer mortality rates among black men in this region (Devesa et al, 1999). Although STDs are more common among men of lower social class (Nakashima et al, 1996; Kilmarx et al, 1997), the relative risks for prostate cancer in our study did not vary by measures of SES, suggesting that the STD-associated risks are not simply an indicator of SES-related factors.

Microbiological and molecular studies to detect infectious agents that may induce prostate cancer have been inconclusive. HPV, which occurs in human prostate cancer and benign prostatic tissue (Cuzick, 1995; IARC, 1995), has been shown to transform human prostate cells in vitro. Seropositivity for HPV-18 and HPV-16 has been associated with subsequent prostate cancer in a Finnish cohort study (Dillner et al, 1998), but a small case–control study of HPV-16 and HPV-11 (Strickler et al, 1998), and our serological investigation of HPV-16 showed little evidence of risk. Since HPV seropositivity is low in these studies (e.g. HPV-16 < 5%), larger investigations will be needed to clarify this relationship. While an excess of prostate cancer has been observed in men with anal cancer, which is linked to HPV infection (IARC, 1995), the epidemiological patterns of HPV-related cervical cancer (Schiffman et al, 1996) are not closely correlated with prostate cancer, although one study reported increased occurrence of cervical cancer in spouses of prostate cancer patients (Feminella and Lattimer, 1974). In addition, no case–control differences have been found with serological responses to herpes simplex, cytomegalovirus and Epstein–Barr virus (Baker et al, 1981; Mandel and Schuman, 1987; Anderson et al, 1996), and studies of HHV8 RNA transcripts in prostate tumour tissue have been inconclusive (Staskus et al, 1997).

Our findings should not be viewed as inconsistent with the widely held view that androgen metabolism plays an important role in prostate carcinogenesis, and that underlying hormonal factors may contribute to the relationship between sexual behaviour, STDs and prostate cancer risk. However, frequency of sexual intercourse was not clearly associated with prostate cancer risk in our study, as one might expect if hormonal factors were responsible for the relationships observed. Furthermore, the association between serum hormone levels and sexual activity among men with normal gonads is uncertain (Buena et al, 1993; Mantzoros et al, 1995; Bagatell and Bremmer, 1997).

Further insight into the relationship between sexual behaviour and prostate cancer, and the mechanisms involved, may come from

*© 2000 Cancer Research Campaign*

prospective epidemiological studies that employ hormonal, microbial and other potential biomarkers of risk. Although a search for infectious agents in unselected prostate tumour tissue has been uninformative to date, study of tumour tissue from men with a history of STDs might prove useful.

In summary, our case–control study of prostate cancer among US blacks and whites revealed excess risks among men who reported a history of gonorrhoea or syphilis, had a positive serology for syphilis, or reported certain sexual behaviours that predispose to these conditions. Although a history of STDs appears associated with a relatively small number of prostate cancers, the findings suggest the need to expand the search for a sexually transmitted agent as well as hormonal and other factors that may contribute to the aetiology of these tumours.

### ACKNOWLEDGEMENTS

The authors thank Ruth Thomson of Westat, Inc., for her assistance in study management and coordination; Stella Sameti and Jerome Mabey of Information Management Systems for computer support; and the interviewers, support staff, physicians, and hospital personnel in the study areas for their participation. The study was funded, in part, through NCI contracts to the Michigan Cancer Foundation (NO1-CP-5109, NO1-CN-05225), the New Jersey State Department of Health (NO1-CP-51089, NO1-CN-31022), the Georgia Center for Cancer Statistics (NO1-CP-51092, NO1-CN-05227), and Westat, Inc. (NO1-CP-51087).

### REFERENCES

Anderson JE, McCormick L and Fichtner R (1994) Factors associated with self-reported STDs – data from a national survey. *Sexually Trans Dis* **21**: 303–308

Anderson S-O, Baron J, Bergstrom R, Lindgren C, Wolk A and Adami H-A (1996) Lifestyle factors and prostate cancer risk: a case–control study in Sweden. *Cancer Epidemiol Biomarkers Prev* **5**: 509–513

Bagatell CJ and Bremmer WJ (1997) Androgens and behavior in men and women. *Endocrinologist* **7**: 97–102

Baker LH, Mebwust WK, Chin TDY, Chapman AL, Hinthorn D and Towle D (1981) The relationship of Herpesvirus to carcinoma of the prostate. *J Urol* **125**: 370–374

Breslow NE and Day NE (1980) *Statistical Methods in Cancer Research. Volume 1. The Analysis of Case–Control Studies*. International Association for Cancer Research: Lyon, France

Brunham RC and Plummer FA (1990) A general model of sexually transmitted disease epidemiology and its implication for control. *Med Clin N Am* **74**: 1339–1352

Buena F, Swerdloff RS, Steiner BS, Lutchmansingh P, Peterson MA, Pandian MR, Galmarini M and Bhasin S (1993) Sexual function does not change when serum testosterone levels are pharmacologically varied within the normal-male range. *Fertil Steril* **59**: 1118–1123

Carter JJ, Koutsky LA, Wipf GC, Christensen ND, Lee S-K, Kuypers J, Kiviat N and Galloway DA (1996) The natural history of human papillomavirus type 16 capsid antibodies among a cohort of university women. *J Infect Dis* **174**: 927–936

Cuzick J (1995) Human papillomavirus infection of the prostate. *Cancer Surveys* **23**: 91–95

Devesa SS, Grauman DJ, Blot WJ, Pennello G, Hoover RN and Fraumeni JF Jr (1999) Atlas of Cancer Mortality in the United States, 1950–94. U.S. Department of Health and Human Services: Rockville, MD

Dillner J, Knekt P, Boman J, Lehtinen M, Af Geijersstam V, Sapp M, Schiller J, Maatela J and Aromaa A (1998) Sero-epidemiological association between human-papillomavirus infection and risk of prostate cancer. *Int J Cancer* **75**: 564–567

Division of STD Prevention (1997) Sexually Transmitted Disease Surveillance, 1996. *Centers for Disease Control and Prevention*: Atlanta, GA

Feminella JJ and Lattimer JR (1974) An apparent increase in genital carcinoma among wives of men with prostatic cancer: an epidemiologic survey. *Pirquet Bull Clin Med* **20**: 3–9

Green LW (1970) Manual for scoring socioeconomic status for research on health behavior. *Publ Health Rep* **85**: 815–827

Haas J, Bolan G, Larsen SA, Clement M, Bacchetti P and Moss A (1990) Sensitivity of treponemal tests for detecting prior treated syphilis during human immunodeficiency virus infection. *J Infect Dis* **162**: 862–866

Hayes RB, Pottern LM, Greenberg R, Schoenberg J, Swanson MG, Liff J, Schwartz AG, Brown LM and Hoover RN (1993) Vasectomy and prostate cancer in US blacks and whites. *Am J Epidemiol* **137**: 263–269

Hayes RB, Pottern LM, Swanson GM, Liff J, Schoenberg J, Greenberg R, Schwartz AG and Hoover RN (1994) Tobacco use and prostate cancer in U.S. blacks and whites. *Cancer Causes Control* **5**: 221–226

Hayes RB, Liff JM, Pottern LM, Greenberg RS, Schoenberg JB, Schwartz AG, Swanson GM, Silverman DT, Brown LM, Hoover RN and Fraumeni JF Jr. (1995) Prostate cancer risk in U.S. blacks and whites with a family history of prostate cancer. *Int J Cancer* **60**: 361–364

Hayes RB, Brown LM, Schoenberg JB, Greenberg RS, Silverman DT, Schwartz AG, Swanson GM, Benichou J, Liff JM, Hoover RN and Pottern L (1997) Alcohol use and prostate cancer risk in U.S. blacks and whites. *Am J Epidemiol* **143**: 692–697

Hayes RB, Ziegler R, Gridley G, Greenberg RS, Swanson GM, Schoenberg JB, Silverman DT, Brown LM, Schwartz AG, Fraumeni JF Jr and Hoover RN (1999) Dietary animal fat and differential risks for prostate cancer among U.S. blacks and whites. *Cancer Epidemiol Biomarkers Prev* **8**: 25–34

Heshmat MY, Kovi J, Herson J, Jones GW, Jackson MA (1975) Epidemiologic association between gonorrhea and prostatic carcinoma. *Urology* **6**: 457–460

Honda GD, Bernstein L, Ross RK, Greenland S, Gerkins V and Henderson BE (1988) Vasectomy, cigarette smoking, and age at first sexual intercourse as risk factors for prostate cancer in middle-aged men. *Br J Cancer* **57**: 326–331

IARC (1995) IARC Monographs on the Evaluation of Carcinogenic Risks to Humans. Volume 64. Human Papillomaviruses. Lyon, France: International Agency for Research on Cancer

Jackson MA, Kovi J, Heshmat MY, Ogunmuyiwa TA, Jones GW, Williams AO, Christian EC, Nkposong EO, Rao MS, Jackson AG and Ahluwalia BS (1980) Characterization of prostatic cancer among blacks: a comparison between a low-incidence area, Ibadan, Nigeria, and a high-incidence area, Washington, DC. *Prostate* **1**: 185–205

Kennedy EJ Jr (1990) Microhemagglutination assay for antibodies to *Treponema pallidum* (MHA-TP). In: *A Manual of Tests for Syphilis*, Larsen SA, Hunter EF and Kraus SJ (eds), pp. 153–166. American Public Health Association: Washington, DC

Kilmarx PH, Zaidi AA, Thomas JC, Nakashima AK, St. Louis ME, Flock ML and Peterman TA (1997) Sociodemographic factors and the variation in syphilis rates among US counties, 1984 through 1993: an ecological analysis. *Am J Publ Health* **87**: 1937–1943

Koopman JS and Longini IM Jr (1994) The ecological effects of individual exposures and nonlinear disease dynamics in populations. *Am J Publ Health* **84**: 836–842

Krain LS (1974) Some epidemiologic variables in prostatic carcinoma in California. *Prev Med* **3**: 154–159

La Vecchia C, Franceschi S, Talamini R, Negri E, Boyle P and D'Avanzo B (1993) Marital status, indicators of sexual activity and prostatic cancer. *J Epidemiol Community Health* **47**: 450–453

Lees REM, Steele R and Wardle D (1985) Arsenic, syphilis, and cancer of the prostate. *J Epidemiol Commun Med* **39**: 227–230

Mandel JS and Schuman LM (1987) Sexual factors and prostatic cancer: results from a case–control study. *J Gerontol* **42**: 259–264

Mantzoros CS, Georgiadis EI and Trichopoulus D (1995) Contribution of dihydrotestosterone to male sexual behavior. *Br Med J* **310**: 1289–1291

Mishina T, Watanabe H, Araki H and Nakao M (1985) Epidemiological study of prostate cancer by matched-pair analysis. *Prostate* **6**: 423–436

Nakashima AK, Rolfs RT, Flock ML, Kilmarx P and Greenspan JR (1996) Epidemiology of syphilis in the United States, 1941–1993. *Sexually Trans Dis* **23**: 16–23

National Cancer Institute (1997) SEER Cancer Statistics Review, 1973–1994. *NIH Publication No.* 97–2789

Nomura AMY and Kolonel LN (1991) Prostate cancer: a current perspective. *Epidemiol Rev* **13**: 200–227

Pienta KJ and Esper PS (1993) Risk factors for prostate cancer. *Ann Intern Med* **118**: 793–803

Romanowski B, Sutherland R, Fick GH, Mooney D and Love EJ (1991) Serologic response to treatment of infectious syphilis. *Ann Intern Med* **114**: 1005–1009

Ross RK and Schottenfeld D (1996) Prostate cancer. In: *Cancer Epidemiology and Prevention*, Schottenfeld D and Fraumeni JF Jr (eds), pp. 1180–1206. Oxford University Press: New York

Ross RK, Shimizu H, Paganini-Hill A, Honda G and Henderson BE (1987) Case–

 *© 2000 Cancer Research Campaign*

control studies of prostatic cancer in blacks and whites in Southern California. *J Natl Cancer Inst* **78**: 869–874

Rotkin ID (1977) Studies in epidemiology of prostatic cancer. *Cancer Treat Rep* **61**: 173–180

Schiffman MH, Bauer HM, Hoover RN, Glass AG, Cadell DM, Rush BB, Scott DR, Sherman ME, Kurman RJ, Wacholder S, Stanton CK and Manos MM (1993) Epidemiologic evidence showing that human papillomavirus infection causes most cervical intraepithelial neoplasia. *J Natl Cancer Inst* **85**: 958–964

Schiffman MH, Brinton LA, Devesa SS and Fraumeni JF Jr (1996) Cervical cancer. In: *Cancer Epidemiology and Prevention*, Schottenfeld D and Fraumeni JF Jr (eds), pp. 1090–1116. Oxford University Press: New York

Staskus KA, Zhong W, Gebhard K, Herndier B, Wang H, Renne R, Beneke J, Pudney J, Anderson DJ, Ganem D and Haase AT (1997) Kaposi's sarcoma-associated herpesvirus gene expression in endothelial (spindle) tumor cells. *J Virol* **71**: 715–719

Steele R, Lees REM, Kraus AS and Rao C (1971) Sexual factors in the epidemiology of the prostate. *J Chron Dis* **24**: 29–37

Strickler HD, Hildesheim A, Viscidi RP, Shah KV, Goebel B, Drummond J, Waters D, Sun Y, Hubbert NL, Wacholder S, Brinton LA, Han C-L, Nasca PC, McLimins R, Turk K, Devairakkam V, Leitman S, Martin C and Schiller JT (1997) Interlaboratory agreement among results of human papillomavirus type 16 enzyme-linked immunosorbent assays. *J Clin Microbiol* **35**: 1751–1756

Strickler HD, Burk R, Shah K, Viscidi R, Jackson A, Pizza G, Bertoni F, Schiller JT, Manns A, Metcalf R, Qu W and Goedert JJ (1998) A multifaceted study of human papillomavirus and prostate carcinoma. *Cancer* **82**: 1118–1125

Strickler HD, Kirk GD, Figuero P, Ward E, Braithwaite AR, Ecoffery C, Drummond J, Goebbel B, Waters D, McLimens R and Manns A (1999) HPV 16 antibody prevalence in Jamaica and the United States reflects differences in cervical cancer rates. *Int J Cancer*

Thomas JC, Schoenbach VJ, Weiner DH, Parker EA and Earp JA (1996) Rural gonorrhea in the southeastern United States: a neglected epidemic? *Amer J Epidemiol* **143**: 269–277

Waksberg J (1978) Sampling methods for random digit dialing. *J Am Stat Assoc* **73**: 40–46

Wynder EL, Mabuchi K and Whitmore WF (1971) Epidemiology of cancer of the prostate. *Cancer* **28**: 344–360

*© 2000 Cancer Research Campaign*

## References

[PDF_00575] Anderson J. E., McCormick L., Fichtner R. (1994). Factors associated with self-reported STDs: data from a national survey.. Sex Transm Dis.

[PDF_00577] Andersson S. O., Baron J., Bergström R., Lindgren C., Wolk A., Adami H. O. (1996). Lifestyle factors and prostate cancer risk: a case-control study in Sweden.. Cancer Epidemiol Biomarkers Prev.

[PDF_00582] Baker L. H., Mebust W. K., Chin T. D., Chapman A. L., Hinthorn D., Towle D. (1981). The relationship of herpesvirus to carcinoma of the prostate.. J Urol.

[PDF_00588] Brunham R. C., Plummer F. A. (1990). A general model of sexually transmitted disease epidemiology and its implications for control.. Med Clin North Am.

[PDF_00590] Buena F., Swerdloff R. S., Steiner B. S., Lutchmansingh P., Peterson M. A., Pandian M. R., Galmarini M., Bhasin S. (1993). Sexual function does not change when serum testosterone levels are pharmacologically varied within the normal male range.. Fertil Steril.

[PDF_00594] Carter J. J., Koutsky L. A., Wipf G. C., Christensen N. D., Lee S. K., Kuypers J., Kiviat N., Galloway D. A. (1996). The natural history of human papillomavirus type 16 capsid antibodies among a cohort of university women.. J Infect Dis.

[PDF_00598] Cuzick J. (1995). Human papillomavirus infection of the prostate.. Cancer Surv.

[PDF_00603] Dillner J., Knekt P., Boman J., Lehtinen M., Af Geijersstam V., Sapp M., Schiller J., Maatela J., Aromaa A. (1998). Sero-epidemiological association between human-papillomavirus infection and risk of prostate cancer.. Int J Cancer.

[PDF_00612] Green L. W. (1970). Manual for scoring socioeconomic status for research on health behavior.. Public Health Rep.

[PDF_00614] Haas J. S., Bolan G., Larsen S. A., Clement M. J., Bacchetti P., Moss A. R. (1990). Sensitivity of treponemal tests for detecting prior treated syphilis during human immunodeficiency virus infection.. J Infect Dis.

[PDF_00627] Hayes R. B., Brown L. M., Schoenberg J. B., Greenberg R. S., Silverman D. T., Schwartz A. G., Swanson G. M., Benichou J., Liff J. M., Hoover R. N. (1996). Alcohol use and prostate cancer risk in US blacks and whites.. Am J Epidemiol.

[PDF_00623] Hayes R. B., Liff J. M., Pottern L. M., Greenberg R. S., Schoenberg J. B., Schwartz A. G., Swanson G. M., Silverman D. T., Brown L. M., Hoover R. N. (1995). Prostate cancer risk in U.S. blacks and whites with a family history of cancer.. Int J Cancer.

[PDF_00617] Hayes R. B., Pottern L. M., Greenberg R., Schoenberg J., Swanson G. M., Liff J., Schwartz A. G., Brown L. M., Hoover R. N. (1993). Vasectomy and prostate cancer in US blacks and whites.. Am J Epidemiol.

[PDF_00620] Hayes R. B., Pottern L. M., Swanson G. M., Liff J. M., Schoenberg J. B., Greenberg R. S., Schwartz A. G., Brown L. M., Silverman D. T., Hoover R. N. (1994). Tobacco use and prostate cancer in blacks and whites in the United States.. Cancer Causes Control.

[PDF_00631] Hayes R. B., Ziegler R. G., Gridley G., Swanson C., Greenberg R. S., Swanson G. M., Schoenberg J. B., Silverman D. T., Brown L. M., Pottern L. M. (1999). Dietary factors and risks for prostate cancer among blacks and whites in the United States.. Cancer Epidemiol Biomarkers Prev.

[PDF_00635] Heshmat M. Y., Kovi J., Herson J., Jones G. W., Jackson M. A. (1975). Epidemiologic association between Gonorrhea and prostatic carcinoma.. Urology.

[PDF_00637] Honda G. D., Bernstein L., Ross R. K., Greenland S., Gerkins V., Henderson B. E. (1988). Vasectomy, cigarette smoking, and age at first sexual intercourse as risk factors for prostate cancer in middle-aged men.. Br J Cancer.

[PDF_00643] Jackson M. A., Kovi J., Heshmat M. Y., Ogunmuyiwa T. A., Jones G. W., Williams A. O., Christian E. C., Nkposong E. O., Rao M. S., Jackson A. G. (1980). Characterization of prostatic carcinoma among blacks: a comparison between a low-incidence area, Ibadan, Nigeria, and a high-incidence area, Washington, DC.. Prostate.

[PDF_00653] Kilmarx P. H., Zaidi A. A., Thomas J. C., Nakashima A. K., St Louis M. E., Flock M. L., Peterman T. A. (1997). Sociodemographic factors and the variation in syphilis rates among US counties, 1984 through 1993: an ecological analysis.. Am J Public Health.

[PDF_00656] Koopman J. S., Longini I. M. (1994). The ecological effects of individual exposures and nonlinear disease dynamics in populations.. Am J Public Health.

[PDF_00658] Krain L. S. (1974). Some epidemiologic variables in prostatic carcinoma in California.. Prev Med.

[PDF_00660] La Vecchia C., Franceschi S., Talamini R., Negri E., Boyle P., D'Avanzo B. (1993). Marital status, indicators of sexual activity and prostatic cancer.. J Epidemiol Community Health.

[PDF_00663] Lees R. E., Steele R., Wardle D. (1985). Arsenic, syphilis, and cancer of the prostate.. J Epidemiol Community Health.

[PDF_00665] Mandel J. S., Schuman L. M. (1987). Sexual factors and prostatic cancer: results from a case-control study.. J Gerontol.

[PDF_00667] Mantzoros C. S., Georgiadis E. I., Trichopoulos D. (1995). Contribution of dihydrotestosterone to male sexual behaviour.. BMJ.

[PDF_00669] Mishina T., Watanabe H., Araki H., Nakao M. (1985). Epidemiological study of prostatic cancer by matched-pair analysis.. Prostate.

[PDF_00671] Nakashima A. K., Rolfs R. T., Flock M. L., Kilmarx P., Greenspan J. R. (1996). Epidemiology of syphilis in the United States, 1941--1993.. Sex Transm Dis.

[PDF_00676] Nomura A. M., Kolonel L. N. (1991). Prostate cancer: a current perspective.. Epidemiol Rev.

[PDF_00678] Pienta K. J., Esper P. S. (1993). Risk factors for prostate cancer.. Ann Intern Med.

[PDF_00680] Romanowski B., Sutherland R., Fick G. H., Mooney D., Love E. J. (1991). Serologic response to treatment of infectious syphilis.. Ann Intern Med.

[PDF_00692] Rotkin I. D. (1977). Studies in the epidemiology of prostatic cancer: expanded sampling.. Cancer Treat Rep.

[PDF_00694] Schiffman M. H., Bauer H. M., Hoover R. N., Glass A. G., Cadell D. M., Rush B. B., Scott D. R., Sherman M. E., Kurman R. J., Wacholder S. (1993). Epidemiologic evidence showing that human papillomavirus infection causes most cervical intraepithelial neoplasia.. J Natl Cancer Inst.

[PDF_00701] Staskus K. A., Zhong W., Gebhard K., Herndier B., Wang H., Renne R., Beneke J., Pudney J., Anderson D. J., Ganem D. (1997). Kaposi's sarcoma-associated herpesvirus gene expression in endothelial (spindle) tumor cells.. J Virol.

[PDF_00705] Steele R., Lees R. E., Kraus A. S., Rao C. (1971). Sexual factors in the epidemiology of cancer of the prostate.. J Chronic Dis.

[PDF_00712] Strickler H. D., Burk R., Shah K., Viscidi R., Jackson A., Pizza G., Bertoni F., Schiller J. T., Manns A., Metcalf R. (1998). A multifaceted study of human papillomavirus and prostate carcinoma.. Cancer.

[PDF_00707] Strickler H. D., Hildesheim A., Viscidi R. P., Shah K. V., Goebel B., Drummond J., Waters D., Sun Y., Hubbert N. L., Wacholder S. (1997). Interlaboratory agreement among results of human papillomavirus type 16 enzyme-linked immunosorbent assays.. J Clin Microbiol.

[PDF_00719] Thomas J. C., Schoenbach V. J., Weiner D. H., Parker E. A., Earp J. A. (1996). Rural gonorrhea in the southeastern United States: a neglected epidemic?. Am J Epidemiol.

[PDF_00724] Wynder E. L., Mabuchi K., Whitmore W. F. (1971). Epidemiology of cancer of the prostate.. Cancer.

